# Prediction of the binding modes between key differential flavor compounds in tobacco leaves at different maturity stages and the olfactory receptor OR1D2

**DOI:** 10.3389/fpls.2026.1894360

**Published:** 2026-10-20

**Authors:** Xianfeng Hu, Wei Xu, Liyuan Zhang, Shenggang Yang, Shouhui Pan, Yan Xue, Maojun Bai, Youxiang Wu, Yichen Yang, Yabo Wang, Yubo Zhang

**Affiliations:** 1 College of Agriculture, Anshun University, Anshun, Guizhou, China; 2 China Tobacco Guizhou Industrial Co Ltd., Guiyang, Guizhou, China; 3 Guizhou Province Tobacco Company Anshun Company, Anshun, Guizhou, China

**Keywords:** tobacco leaves maturity, microstructure, olfactory receptor, untargeted metabolomics, Yunyan 87

## Abstract

**Introduction:**

Leaf maturity is a critical determinant of the sensory quality of tobacco. To elucidate the microstructural characteristics of Yunyan 87 tobacco at distinct maturity stages and the perception mechanisms of key differential flavor compounds mediated by the olfactory receptor OR1D2.

**Methods:**

This study combined scanning electron microscopy (SEM) and untargeted metabolomics with molecular docking and molecular dynamics (MD) simulations to elucidate the binding mechanisms of key flavor compounds to the olfactory receptor OR1D2.

**Results:**

The results indicated that as maturity progressed, the epidermal cell arrangement transitioned from compact to loose, accompanied by an increase in intercellular spaces. Concurrently, the surface microstructure evolved from gentle striations at the unripe stage to distinct ridges or rugae at the mature and overripe stages. Metabolomic analysis revealed that 226 metabolites were identified in both unripe (QS) and mature (GS) leaves, whereas the number increased to 233 in overripe (CS) leaves. A total of 10 differentially abundant metabolites were screened across the three groups. Notably, the contents of key aroma compounds including γ-valerolactone, benzaldehyde, β-cyclocitral, and isopentenol were significantly higher in mature leaves compared to unripe and overripe samples. Molecular docking analysis demonstrated significant variations in binding affinity between the 10 differential flavor compounds and the OR1D2 receptor, with β-cyclocitral exhibiting the strongest binding energy. Molecular dynamics simulation further confirmed the stable binding complex, revealing that the interaction was primarily driven by van der Waals forces, supplemented by electrostatic and hydrophobic interactions.

**Discussion:**

Collectively, these findings indicate that harvesting Yunyan 87 at the mature stage is optimal for maximizing quality. This study provides a theoretical foundation for the precise regulation of harvesting maturity in tobacco production.

## 1 Introduction

Tobacco (*Nicotiana tabacum* L.) is a globally significant economic crop, contributing substantially to the economies of numerous countries (Li et al., 2021; Hu et al., 2025; Xiao et al., 2025). Yunyan 87 is one of the major cultivated tobacco varieties in China, with extensive planting area and strong representativeness. Yunyan 87 leaves are of high quality and exhibit excellent industrial usability, making it a staple flue-cured tobacco variety for Chinese-style cigarettes for a long time. Aroma precursors are compounds in tobacco leaves characterized by minimal intrinsic aroma. Through enzymatic, non-enzymatic, or pyrolytic transformations during curing, fermentation, or smoking, they convert into volatile aroma compounds, serving as the fundamental material determinant of tobacco leaf quality (Geng et al., 2023). The quality attributes of tobacco directly determine the sensory properties and industrial applicability of cigarette products (Liu et al., 2022). Tobacco leaf chemical profile is a key indicator for assessing tobacco product quality (Tsaballa et al., 2020). Tobacco leaf maturity directly influences yield, economic value, and the sensory quality of processed products (Chen et al., 2021). The spatial distribution of compounds in tobacco leaves across different growth stages further offers critical signals for identifying the optimal harvest time (Fernando et al., 2013; Abdollahi et al., 2020). Tobacco leaf age significantly affects tissue intercellular spaces, while both leaf age and curing stages markedly influence the upper epidermis, palisade tissue, spongy tissue, and leaf thickness. Tobacco leaves harvested at optimal maturity stages demonstrate reduced membrane lipid peroxidation, well-balanced chemical compositions, and enhanced economic value, thereby improving overall usability (Chen et al., 2019). However, research on the microscopic structural characteristics of Yunyan 87 middle-leaf tobacco at different maturity stages and the perception mechanisms of the olfactory receptor toward key differential flavor compounds remains limited.

The metabolome comprises the complete set of small-molecule metabolites within a biological organism, including those involved in cellular metabolic regulation and their dynamic variations (Maia et al., 2023). As an essential component of systems biology, metabolomics employs high-throughput techniques to qualitatively and quantitatively analyze these metabolites, thereby revealing metabolic networks and their associations with genes and the environment (Li et al., 2024). The metabolome enables the simultaneous analysis of thousands of metabolites, providing a global perspective on the metabolic status of an organism and directly reflecting the downstream effects of gene expression and protein activity, thereby addressing the limitations of genomic and transcriptomic data (Silva et al., 2021; Shen et al., 2023). Metabolomics has been extensively applied to profile the key aroma-active constituents of tobacco (Jia et al., 2025). Nevertheless, the perception of tobacco flavor depends not only on the phytochemical profile of the leaves but, more critically, on the recognition and decoding of these volatile molecules by the human olfactory apparatus. The core mechanism of olfactory perception lies in the specific binding of odorant molecules to olfactory receptors (ORs) and the subsequent signal transduction. Within this framework, the class II subfamily of G protein-coupled receptors (GPCRs) represents a critical functional branch of the human olfactory receptor family (Gil et al., 2026). Tobacco leaves contain abundant volatile compounds such as pyrazines, aldehydes, and terpenes, which are known to exhibit affinity for the human olfactory receptor OR1D2. Moreover, OR1D2 possesses a relatively broad ligand-binding profile and its structural information is comparatively well characterized (Chen et al., 2026; Gil et al., 2026). Furthermore, its three-dimensional structure or homology model can be predicted with high confidence using tools such as AlphaFold, which provides a reliable structural basis for molecular docking and molecular dynamics simulations. Molecular docking studies have demonstrated that OR1D2 exhibits binding free energies lower than -6 kcal/mol with five key floral odorants, achieving stable complex formation primarily through hydrogen bonding and hydrophobic interactions (Hu et al., 2026). Chen et al. identified OR1D2 as the primary target for pyrazine compounds key aroma-active components generated during the roasting of Longjing tea (Chen et al., 2026). Furthermore, Ji et al. reported that OR1D2 displays a preferential binding affinity for umami-like odorants; the transmembrane helices TM3, TM5, and TM6 were identified as crucial binding domains, where hydrogen bonds and hydrophobic forces constitute the core driving forces (Ji et al., 2025). The formation of tobacco leaf flavor also relies on the synergistic effects of volatile aroma components; at its essence, perception is similarly governed by the specific binding of aroma molecules to olfactory receptors and the subsequent activation of signaling pathways.

In this study, based on the pivotal role of leaf maturity in determining tobacco quality. This study proposes the central scientific hypothesis that variations in tobacco leaf maturity significantly affect its microstructure and the accumulation of characteristic flavor metabolites. These changes subsequently modulate the composition and content of key aroma molecules, thereby regulating their specific binding affinity to the olfactory receptor OR1D2. Using middle leaves of Yunyan 87 at different maturity stages as materials, this study integrates scanning electron microscopy (SEM) and untargeted metabolomics to systematically characterize the dynamic changes in leaf microstructure and metabolic profiles, respectively. Furthermore, molecular docking and molecular dynamics (MD) simulations were employed to predict the binding modes and stability of key flavor compounds with OR1D2. This research aims to elucidate the intrinsic relationships among maturity, microstructure, metabolic characteristics, and olfactory receptor responses, thereby providing a theoretical foundation for optimizing harvesting criteria.

## 2 Materials and methods

### 2.1 Experimental materials

The electron microscopy fixative and phosphate-buffered saline (PBS) were purchased from Servicebio (Wuhan, China). Absolute ethanol and isoamyl acetate were sourced from Sinopharm Chemical Reagent Co., Ltd. (China), while osmium tetroxide was obtained from Ted Pella Inc. (USA).

The experiment was carried out at the experimental field of Getuyuhe Town, Ziyun County, Anshun City, Guizhou Province (25.67756°N, 106.16586°E). The tested tobacco variety was Yunyan 87, which was transplanted on April 21, and subjected to conventional cultivation and management practices. Basal fertilizer consisted of a special compound fertilizer (N:P_2_O_5_:K_2_O = 10:11:24), applied at a rate of 35 kg per mu. Topdressing was performed using water-soluble fertilizers, including No. 1 (1.25 kg per mu), No. 2 (9 kg per mu), and No. 3 (10 kg per mu). Additionally, calcium magnesium phosphate fertilizer was applied at 25 kg per mu, and potassium sulfate was supplemented at 10 kg per mu. Furthermore, 80 kg per mu of organic fertilizer and 30 kg per mu of oilseed meal were also applied. Middle leaves were selected as experimental materials and classified into three maturity stages: underripe (QS, 39 ≤ SPAD value ≤ 47), mature (CS, 26 ≤ SPAD value ≤ 30), and overripe (GS, 13 ≤ SPAD value ≤ 17) (Zheng et al., 2020). Each maturity stage treatment included three biological replicates. Following harvesting at different maturity stages and subsequent drying, the collected tobacco leaf samples underwent untargeted metabolomics analysis by Suzhou Panomike Biomedical Technology Co., Ltd.

### 2.2 Scanning electron microscopy observation

Sample preparation and fixation: Fresh tissues were rapidly collected from predefined locations within 1–3 minutes, with tissue blocks limited to a maximum size of 3 mm^2^. The sample surfaces were gently rinsed with PBS to protect the scanning surfaces, which were then properly marked. Samples were immediately fixed with 2.5% glutaraldehyde at room temperature for 2 h, and subsequently stored at 4 °C. Post-fixation: The fixed samples were rinsed three times (15 min each) with 0.1 M phosphate buffer (PB, pH 7.4). Subsequently, the samples were post-fixed in 1% osmium tetroxide (prepared in 0.1 M phosphate buffer, PB, pH 7.4) for 1–2 h at room temperature in the dark. After post-fixation, the samples were rinsed again three times (15 min per rinse) with 0.1 M phosphate buffer (PB, pH 7.4). Dehydration: Tissues were sequentially dehydrated in 30%, 50%, 70%, 80%, 90%, 95%, and 100% ethanol (15 min each concentration), followed by 15 min in acetone. Drying: The samples were placed in a critical point dryer (CPD300, Quorum Technologies Limited) for drying. Sample conductivity treatment: The samples were mounted on conductive carbon film double-sided adhesive tape and placed on the sample stage of an ion sputter coater (MC1000, Hitachi, Ltd.) for gold sputtering for approximately 30 s. Observation and imaging: Representative fields of view, reflecting the general structural characteristics of the tissue at a consistent magnification, were selected and imaged using a scanning electron microscope (SU8100, Hitachi, Ltd.). For density quantification of stomata and glandular trichomes, SEM images were analyzed using ImageJ software (Version 1.53e, NIH, USA). Pixel calibration was performed based on the scale bar. A standardized region with a fixed area (A) was selected at the center of each field of view. Only glandular trichomes with intact basal structures and stomata with clearly defined guard cell contours were counted (N). Density was calculated using the formula Density = N/A. For each sample, at least five random fields of view were analyzed, and the mean density was reported. Three biological replicates (leaves) were examined per maturity stage.

### 2.3 Untargeted metabolomics analysis

#### 2.3.1 Sample storage and pretreatment

Following harvest, tobacco leaves were immediately snap-frozen in liquid nitrogen and stored at −80 °C until further analysis. Frozen samples were retrieved from the −80 °C freezer, ground under liquid nitrogen protection, and thoroughly vortex-mixed to ensure homogeneity. Approximately 500 mg of ground powder from each biological replicate was accurately weighed and transferred into a dedicated headspace vial. Subsequently, 2 mL of saturated NaCl solution and 20 μL of internal standard solution (10 μg/mL) were sequentially added to each vial for quantitative calibration.

#### 2.3.2 Solid-phase microextraction conditions​

The analysis was performed using a CTC CombiPAL autosampler with a 50/30 μm DVB/CAR/PDMS fiber coating. The extraction was conducted at 50 °C for 30 min under continuous shaking at 250 rpm for the initial 15 min, followed by static extraction for the remaining 15 min. Desorption was carried out for 5 min prior (Masite et al., 2022).

#### 2.3.3 Instrumental detection​

Gas chromatography-mass spectrometry (GC-MS) analysis was performed using an Agilent Technologies (USA) 7890B-5977B GC-MS system, consisting of an Agilent 7890B gas chromatograph coupled to an Agilent 5977B mass spectrometer. A 2 mL sample was transferred into a headspace vial and sealed with a cap. Chromatographic separation was performed using a DB-WAX column (30 m × 0.25 mm × 0.25 μm). The injection temperature was set at 260 °C with splitless injection. Helium gas (99.999%) was used as the carrier gas at a flow rate of 1 mL/min. The oven temperature program was: initial hold at 40 °C for 5 min, followed by a ramp of 5 °C/min to 220 °C, then 20 °C/min to 250 °C with a final hold at 2.5 min. The interface temperature was maintained at 260 °C, with the ion source at 230 °C and quadrupole at 150 °C. Electron ionization (EI+) at 70 eV was employed, using full scan mode with a mass range of 20-400. Mass spectra were acquired against the NIST2014 spectral library (Sei et al., 2021).

### 2.4 Molecular docking and molecular dynamics simulation

#### 2.4.1 Molecular docking

The structural information for the olfactory receptor OR1D2 was retrieved from the UniProt database (https://www.uniprot.org/). The structural model, predicted by AlphaFold, was processed to retain only the protein backbone and side-chain atoms essential for docking, while ligands, water molecules, and non-essential ions were removed. Subsequently, the receptor was preprocessed using the prepare_receptorscript within MGLTools 1.5.7 to add hydrogen atoms and calculate Gasteiger charges, followed by conversion to the PDBQT format compatible with AutoDock Vina. Three-dimensional structures of candidate compounds were obtained from the PubChem database. These ligands were similarly preprocessed using MGLTools 1.5.7, involving hydrogen addition, charge assignment, and the definition of rotatable bonds, prior to conversion into PDBQT format. To ensure accurate binding mode prediction, a grid box was specifically defined to encompass the predicted orthosteric binding pocket of OR1D2, rather than the entire protein surface. The grid box was centered at coordinates center_x = 5.123, center_y = 3.449, and center_z = -10.462, with dimensions set to size_x = 30 Å, size_y = 30 Å, and size_z = 30 Å. Molecular docking was performed using AutoDock Vina 1.1.2 with an exhaustiveness parameter of 8, num_modes set to 20, and an energy_range of 3, utilizing 15 CPUs for parallel computation. The binding pose with the lowest binding energy for each ligand was selected for subsequent molecular dynamics simulations and interaction analysis.

#### 2.4.2 Molecular dynamics simulation

Based on the protein–ligand complex obtained from molecular docking, the all-atom protein–ligand–POPC bilayer system was constructed using CHARMM-GUI Membrane Builder v3.7 while preserving the docked ligand conformation. The system comprised one 303-residue protein, one neutral ligand, 201 POPC molecules, 16,771 CHARMM-modified TIP3P water molecules, 44 K^+^ ions, and 56 Cl^-^ ions, yielding a salt concentration of approximately 0.15 mol·L^-^¹. The initial periodic box dimensions were 9.0215 × 9.0215 × 10.9649 nm³, with a total of 82,306 atoms. The protein, membrane lipids, and ligand were described by the CHARMM36m, CHARMM36 lipid, and CGenFF force fields, respectively, wherein the CGenFF program and parameter versions were 4.0 and 5.0. Following energy minimization using the steepest descent method, the system underwent two-stage NVT equilibration and four-stage NPT equilibration, with a total equilibration time of 1.875 ns, during which positional and dihedral restraints on the protein, ligand, and membrane lipids were gradually released. Production simulations were performed using GROMACS 2022.2 on an NVIDIA RTX PRO 6000 GPU at 310.15 K and 1 bar, with a time step of 2 fs and a total simulation time of 100 ns. Temperature was controlled using the velocity-rescaling method, and pressure was coupled semi-isotropically using the C-rescale method. Long-range electrostatic interactions were treated with the PME method using an electrostatic cutoff of 1.2 nm, while Lennard–Jones interactions were smoothly truncated between 1.0 and 1.2 nm. Hydrogen-involved covalent bonds were constrained using the LINCS algorithm. Trajectories were output every 20 ps. Post-simulation structural and interaction analyses were conducted using GROMACS tools and VMD/PyMOL. The binding free energy of the β-cyclocitral–OR1D2 complex was then estimated using the MM–PBSA method implemented in gmx_MMPBSA. The calculations were performed with an ionic concentration of 0.15 M, a solute dielectric constant of 4, and a solvent dielectric constant of 78.54 to simulate physiological conditions.

### 2.5 Statistical analysis

Analysis of variance (ANOVA) was carried out using Statistix 8.1 (Tallahassee, FL, USA). The mass spectrometry data analysis process involved sequential execution of key processing steps including peak identification, peak alignment, and normalization. The processed data were comprehensively compared with established metabolite databases or reference standards to achieve both qualitative and quantitative analysis of metabolites in the samples. All multivariate and network analyses including Principal component analysis (base R 4.1.2, log2 transformation and zero centering), heatmap generation (ComplexHeatmap 2.9.4, log2 transformation and UV scaling), sample correlation plotting (corrplot 0.92), OPLS-DA (MetaboAnalystR 1.0.1, log2 transformation and UV scaling) and correlation network construction (igraph 1.2.11) were performed following the standardized metaboanalyst workflow, and all software versions and key parameters were fully documented to ensure methodological transparency and reproducibility. Differential metabolites were screened based on the following criteria: *p*-value ≤ 0.05 and VIP ≥ 1, one-way ANOVA *p*-value ≤ 0.05.

## 3 Results and analysis

### 3.1 Microstructure of tobacco leaves at different maturity stages

To clarify the microstructural variations in Yunyan 87 middle leaves across distinct maturity stages (underripe, mature, and overripe), SEM was employed to analyze dry leaf samples from each stage. As illustrated in **Figure 1**, Stomatal density was significantly higher in mature leaves (58.94 ± 4.155 stomata/mm^2^) than in underripe leaves (47.39 ± 1.676 stomata/mm^2^; *p* < 0.05), with no significant differences observed between overripe (51.51 ± 2.356 stomata/mm^2^) and the other two stages (*p* > 0.05). Underripe leaf samples were characterized by tightly arranged epidermal cells, minimal intercellular spaces, and smooth surface striations. In contrast, mature and overripe leaf samples exhibited enlarged intercellular spaces, distinctly patterned surface striations, and even ridged protrusions.

**Figure 1:**
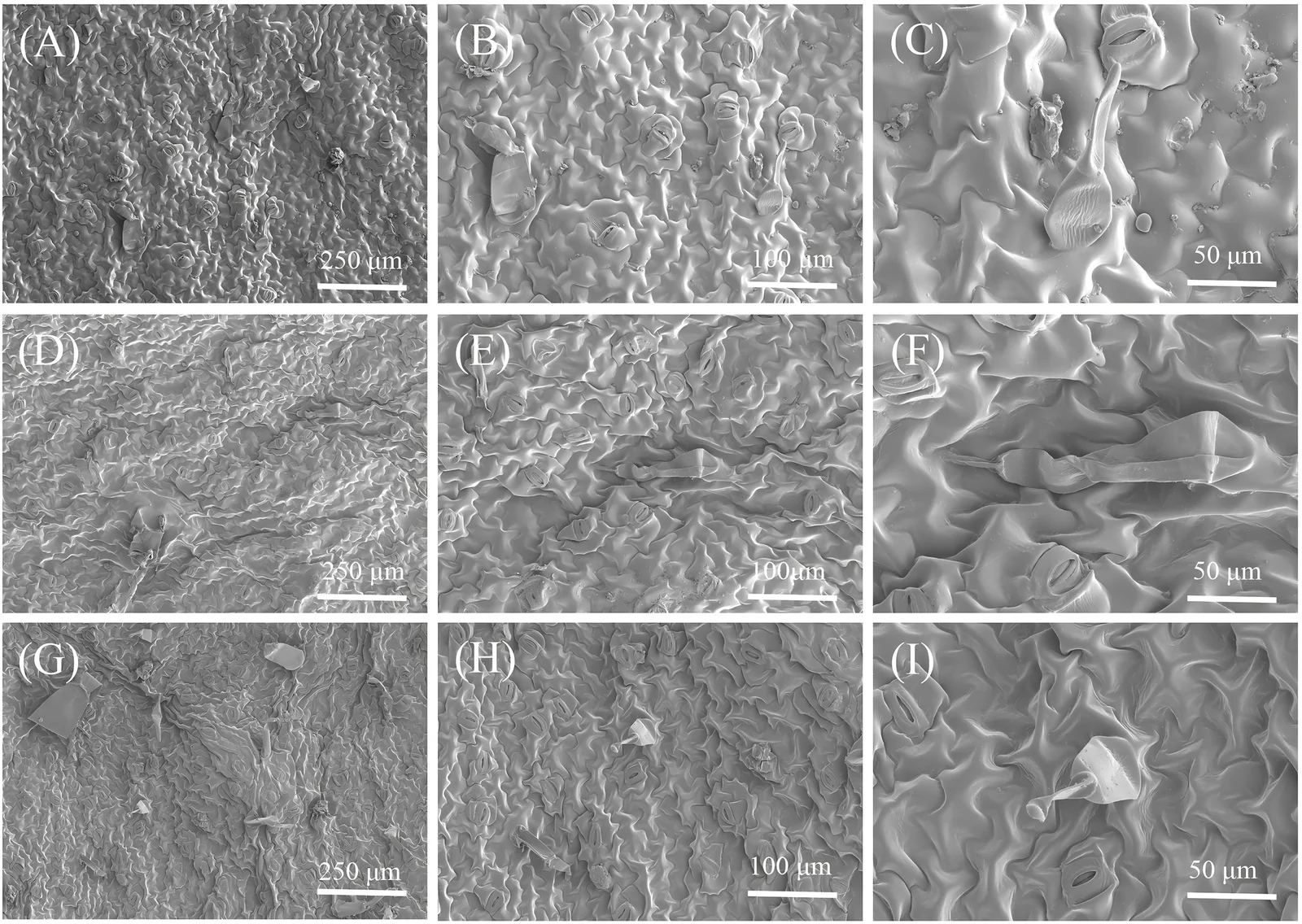
Microstructure of Yunyan 87 middle leaves at different maturity stages. **(A–C)** Underripe tobacco leaves; **(D–F)** mature tobacco leaves; **(G–I)** overripe tobacco leaves.

### 3.2 Untargeted metabolomic analysis of tobacco leaves with different maturities

#### 3.2.1 Cluster analysis of tobacco leaf at different maturity stages

Cluster analysis was performed to group metabolite data from tobacco leaves at different maturity stages, thereby identifying clusters of metabolites with similar metabolic patterns. As depicted in **Figure 2**, the cluster analysis results of tobacco leaf samples at different maturity stages revealed that QS3, QS1, CS1, and QS2 clustered together as a distinct group, whereas CS3, GS1, CS2, and GS2 formed a separate cluster. Notably, the mature middle leaves of Yunyan 87 were located in the transitional region between the underripe and mature stages, exhibiting metabolic similarities to both underripe and mature leaf samples. By identifying clusters of metabolites with similar metabolic characteristics, this approach provides a basis for understanding the metabolic regulatory mechanisms during tobacco maturation.

**Figure 2:**
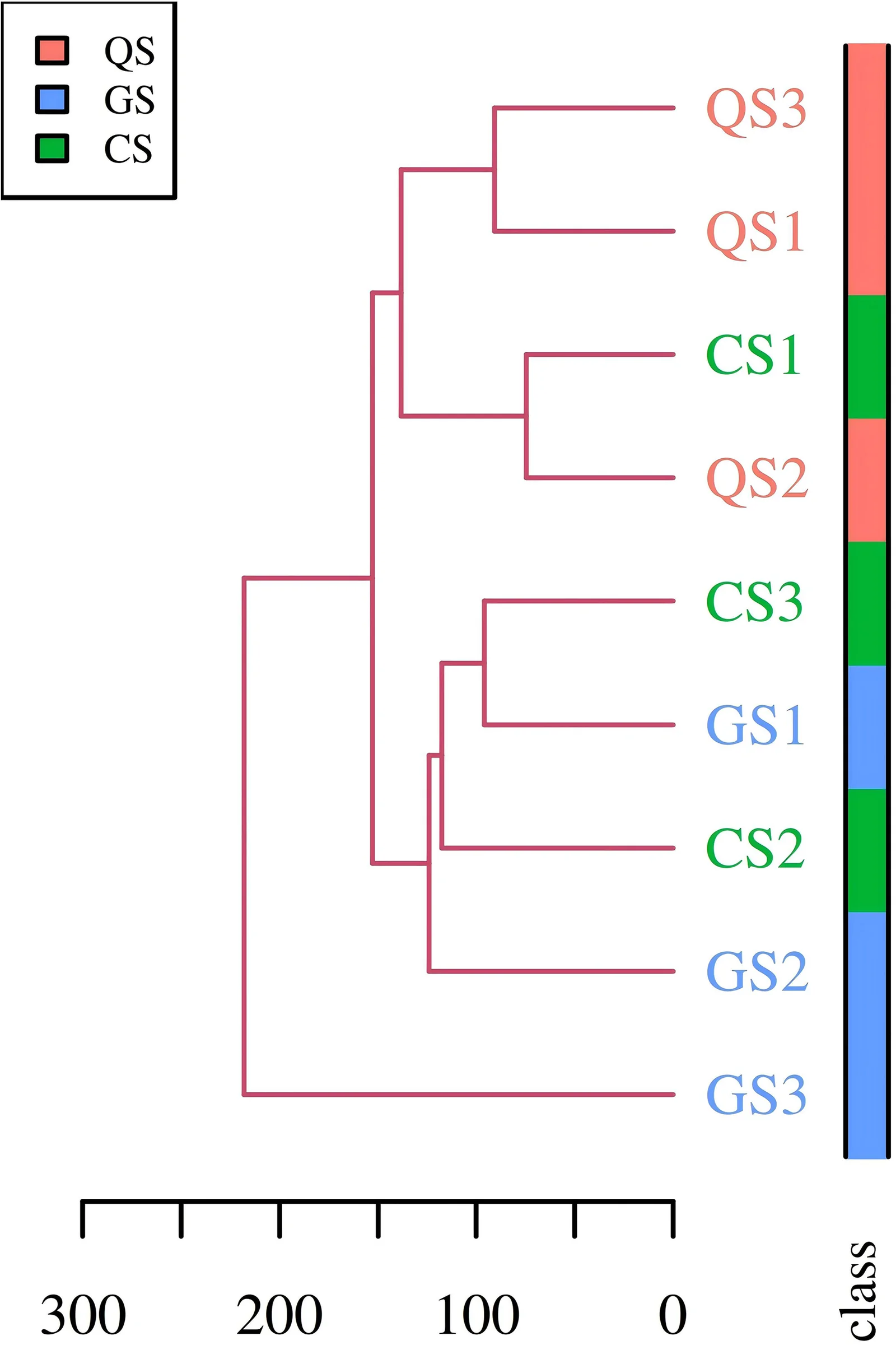
Cluster analysis of tobacco leaf samples at different maturity stages. QS, Underripe; CS, Mature; GS, Overripe.

#### 3.2.2 Multivariate statistical analysis

Multivariate statistical analysis of the non-targeted metabolomics data revealed that both the PCA loading plot (**Figure 3A**) and the PLS-DA model (**Figure 3B**) effectively characterized the primary metabolic disparities among sample groups. The first two principal components individually explained 48.3% and 43.2% of the total variance, respectively, with a cumulative contribution of 91.5%. The loading plot indicated that the majority of metabolites exhibited relatively stable expression across groups; however, variables located on the negative side of PC1 with high loading values were identified as key contributors driving group separation and were regarded as potential differential biomarkers. Furthermore, permutation testing of the PLS-DA model (**Figure 3C**) yielded a Q^2^Y intercept of −0.09 (less than 0.05 and negative), confirming the absence of overfitting.

**Figure 3:**
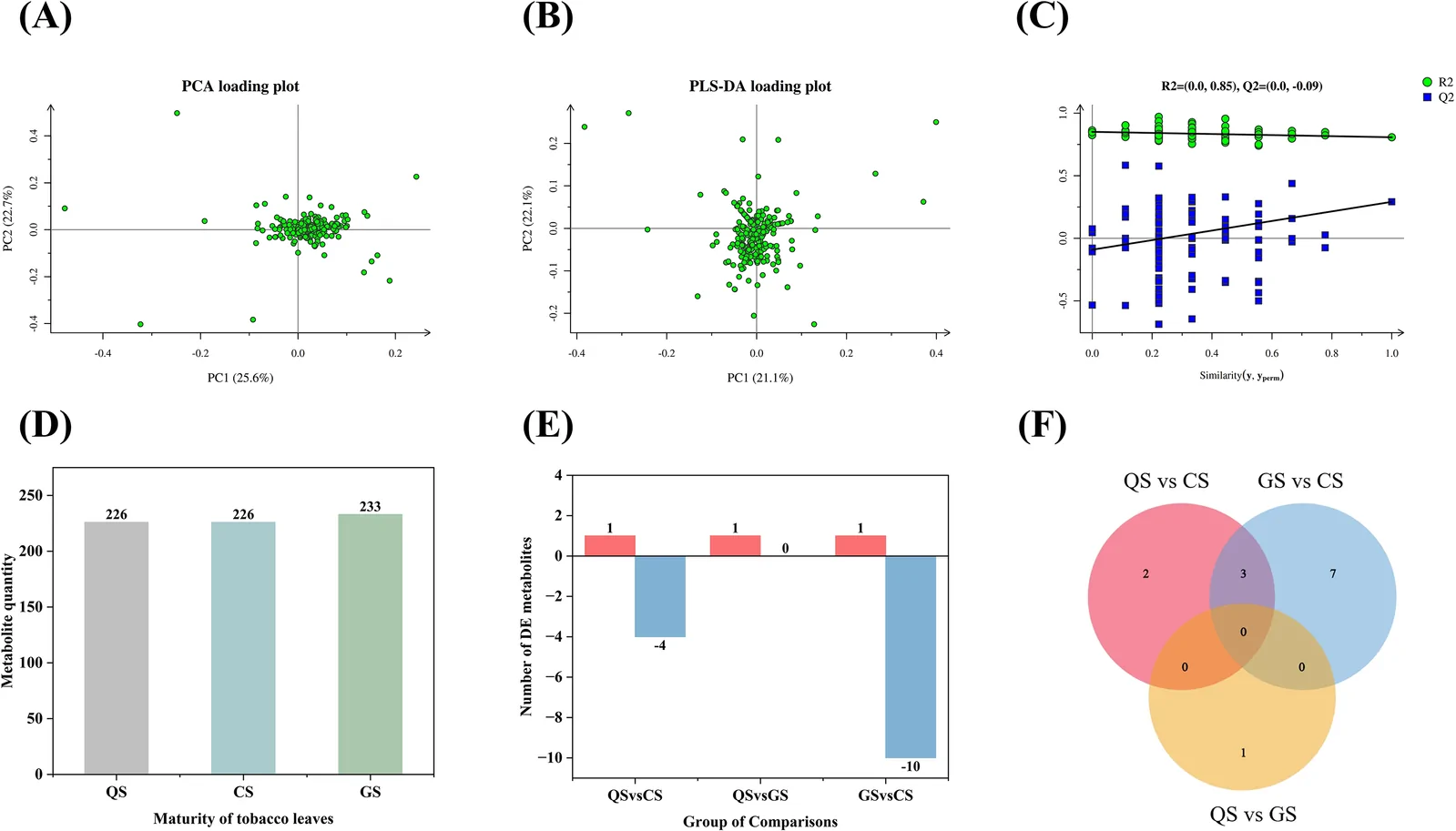
Metabolomics analysis of tobacco leaves at different maturity stages. **(A)** Principal component analysis (PCA) score plot; **(B)** partial least squares discriminant analysis (PLS-DA) score plot; **(C)** permutation test plot; **(D)** bar chart showing the number of differential metabolites; **(E)** statistics of the number of differential metabolites between groups; **(F)** venn diagram of differential metabolites among the three groups. QS, Underripe; CS, Mature; GS, Overripe.

#### 3.2.3 Statistical analysis of differential metabolites

Metabolomic profiling of dried tobacco leaves collected at distinct maturity stages identified 226 metabolites in both underripe and mature samples, whereas 233 metabolites were detected in overripe samples (**Figure 3D**). As illustrated in **Figure 3E**, comparative analysis revealed that: (i) compared to mature leaves, underripe leaves had 1 upregulated and 4 downregulated metabolites, (ii) compared to overripe leaves, underripe leaves exhibited 1 upregulated and 0 downregulated metabolites, and (iii) compared to mature leaves, overripe leaves showed 1 upregulated and 10 downregulated metabolites. The Venn diagram of differential metabolites (**Figure 3F**) further showed that three metabolites were commonly identified in both the underripe *vs*. mature and overripe *vs*. mature comparisons.

#### 3.2.4 Hierarchical clustering analysis of differential metabolites

The dataset was standardized using the pheatmap package in R to construct a hierarchical clustering heatmap of metabolite relative quantitative values (**Figure 4A**). In this visualization, relative abundance levels are represented by color gradients, with columns corresponding to samples and rows representing metabolites. The clustering heatmap revealed that mature tobacco leaves exhibited upregulated differential metabolites including (4S)-4-methylhex-1-ol, β-cyclocitral, trans-crotonic acid, isoprenol, 3-hydroxy-2-butanone, 3-methylbenzyl alcohol, γ-valerolactone, and benzaldehyde, while downregulated metabolites primarily comprised isocrotonic acid and 1-methyl-2-pyrrolidinone, when compared to underripe and overripe leaves.

**Figure 4:**
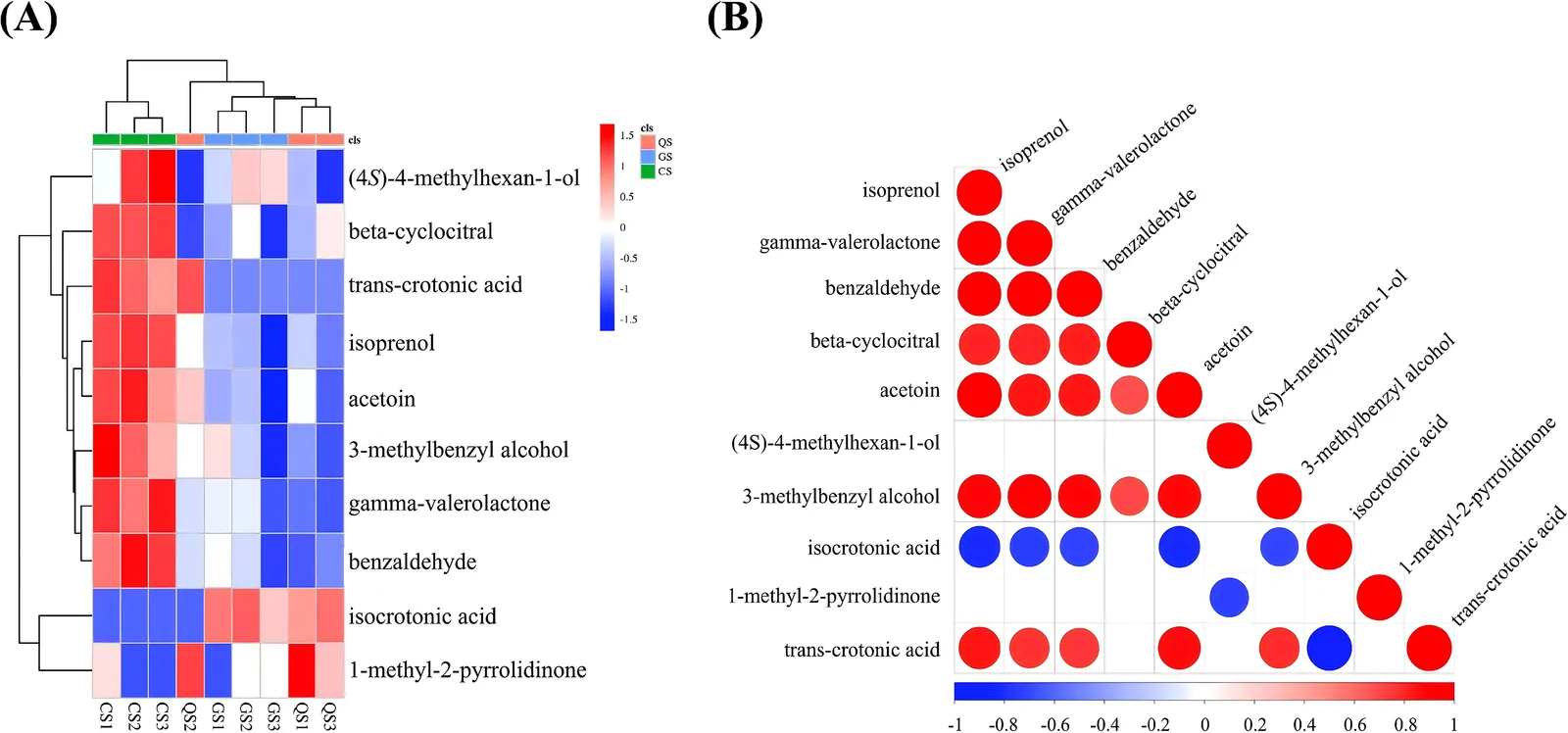
Hierarchical clustering heatmap **(A)** and correlation matrix bubble plot of metabolites **(B)**.

#### 3.2.5 Relative content analysis of differential metabolites

The relative contents of 10 key differential metabolites in tobacco leaves at different maturity stages were analyzed. As illustrated in **Figure 5**, mature tobacco leaf samples exhibited significantly higher relative levels of (4*S*)-4-methylhex-1-ol, β-cyclocitral, trans-crotonic acid, isoprenol, 3-hydroxy-2-butanone, 3-methylbenzyl alcohol, γ-valerolactone, and benzaldehyde compared to both underripe and overripe samples. In contrast, isocrotonic acid and 1-methyl-2-pyrrolidinone exhibited relatively lower abundances in mature leaves compared to the other two maturity stages.

**Figure 5:**
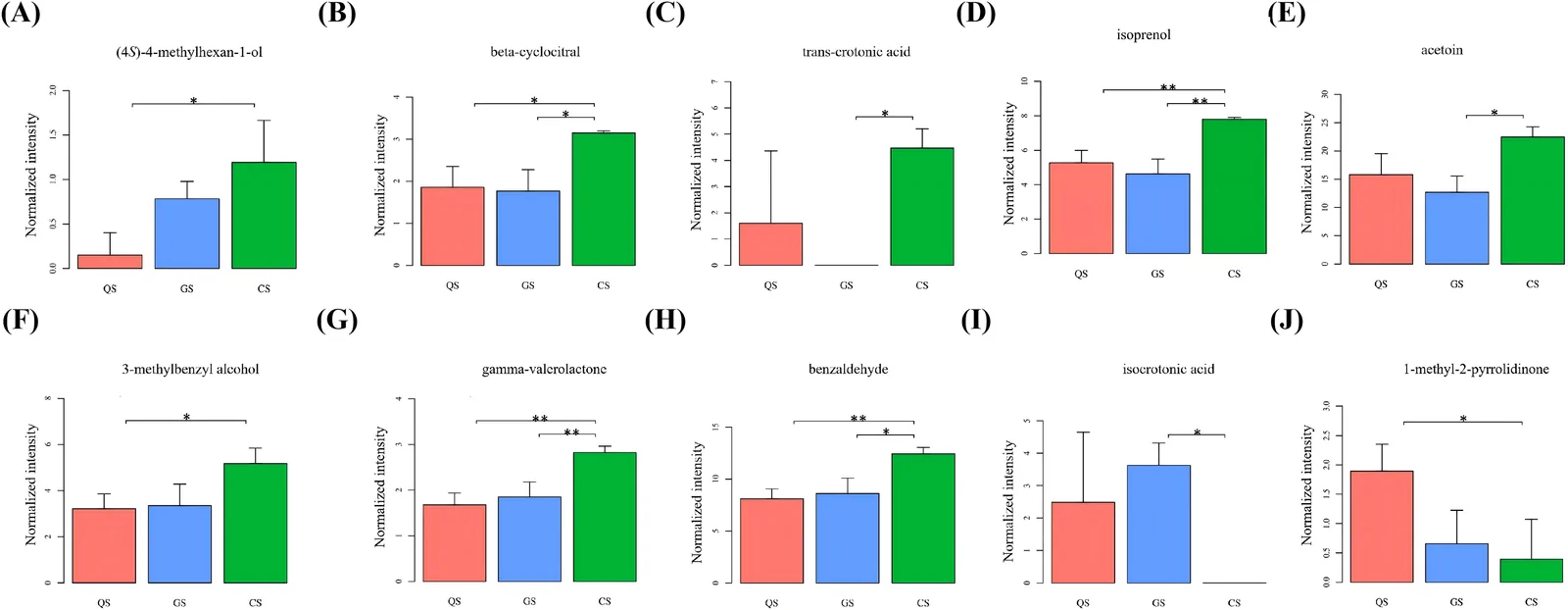
Relative content analysis of differential metabolites. **(A)** (4S)-4-methylhexan-1-ol; **(B)** beta-cyclocitral; **(C)** trans-crotonic acid; **(D)** isoprenol; **(E)** acetoin; **(F)** 3-methylbenzyl alcohol; **(G)** gamma-valerolactone; **(H)** benzaldehyde; (I) isocrotonic acid; (J) 1-methyl-2-pyrrolidone. QS, underripe; CS, mature; GS, overripe. Values are normalized intensities. Asterisks indicate significant differences: *p < 0.05; **p < 0.01.

#### 3.2.6 Correlation analysis of differential metabolites

To reveal the interactions among metabolites, this study analyzed the correlations in their variation trends by calculating Pearson correlation coefficients among differentially abundant metabolites. As illustrated in **Figure 4B**, isocrotonic acid displayed negative correlations with isoprenol, γ-valerolactone, benzaldehyde, 3-hydroxy-2-butanone, and 3-methylbenzyl alcohol, whereas 1-methyl-2-pyrrolidinone exhibited a negative correlation with (4S)-4-methylhex-1-ol. In contrast, trans-crotonic acid displayed positive correlations with isoprenol, γ-valerolactone, benzaldehyde, 3-hydroxy-2-butanone, and 3-methylbenzyl alcohol.

### 3.3 Prediction and molecular dynamics simulation

#### 3.3.1 Prediction of ligand binding to receptor OR1D2

This heatmap visually illustrates the differences in binding affinity between ten ligands and the receptor OR1D2 (**Figure 6**). All binding energy values are negative, with a larger absolute value indicating stronger binding. The results show that β-cyclocitral exhibits the strongest binding affinity (-6.2 kcal/mol), followed by 3-methylbenzyl alcohol (-5.8 kcal/mol). In contrast, 3-hydroxy-2-butanone shows the weakest binding affinity (-3.8 kcal/mol). The gradient shift in color from deep red to pale yellow clearly reflects the gradual decrease in ligand binding strength.

**Figure 6:**
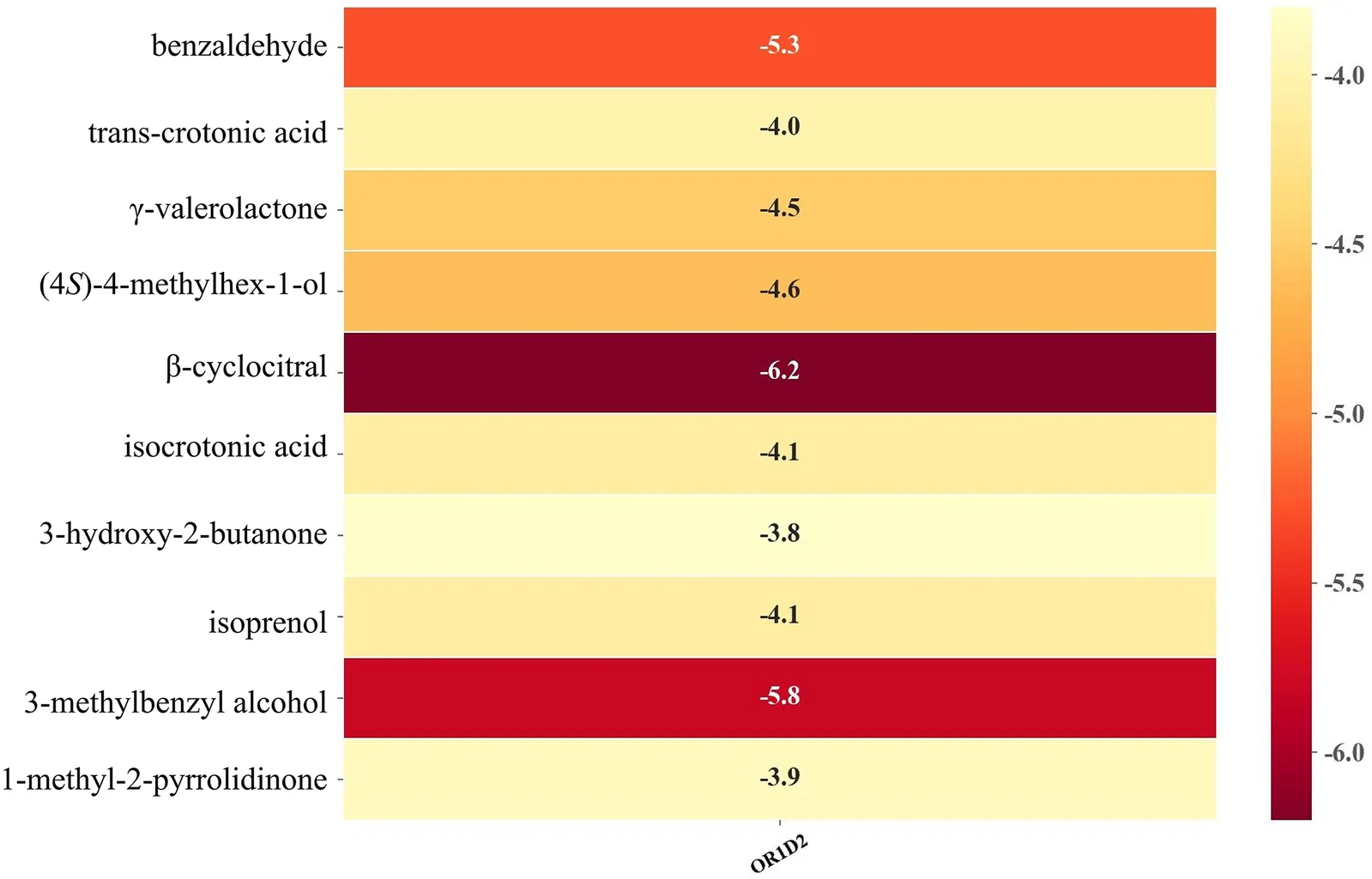
Heatmap of binding energies between small molecules and the OR1D2 receptor.

As shown in **Figure 7D**, β-cyclocitral is primarily embedded in a hydrophobic cavity of OR1D2. Van der Waals interactions are observed with TYR155, ILE200, GLY203, CYS204, and LEU208. Additionally, alkyl/Pi-alkyl hydrophobic contacts are identified with LEU199, PHE207, and LEU255, while a Pi-Sigma interaction is evident between TYR259 and the ligand. These interactions indicate that the overall binding architecture is predominantly stabilized by hydrophobic effects and van der Waals forces.

**Figure 7:**
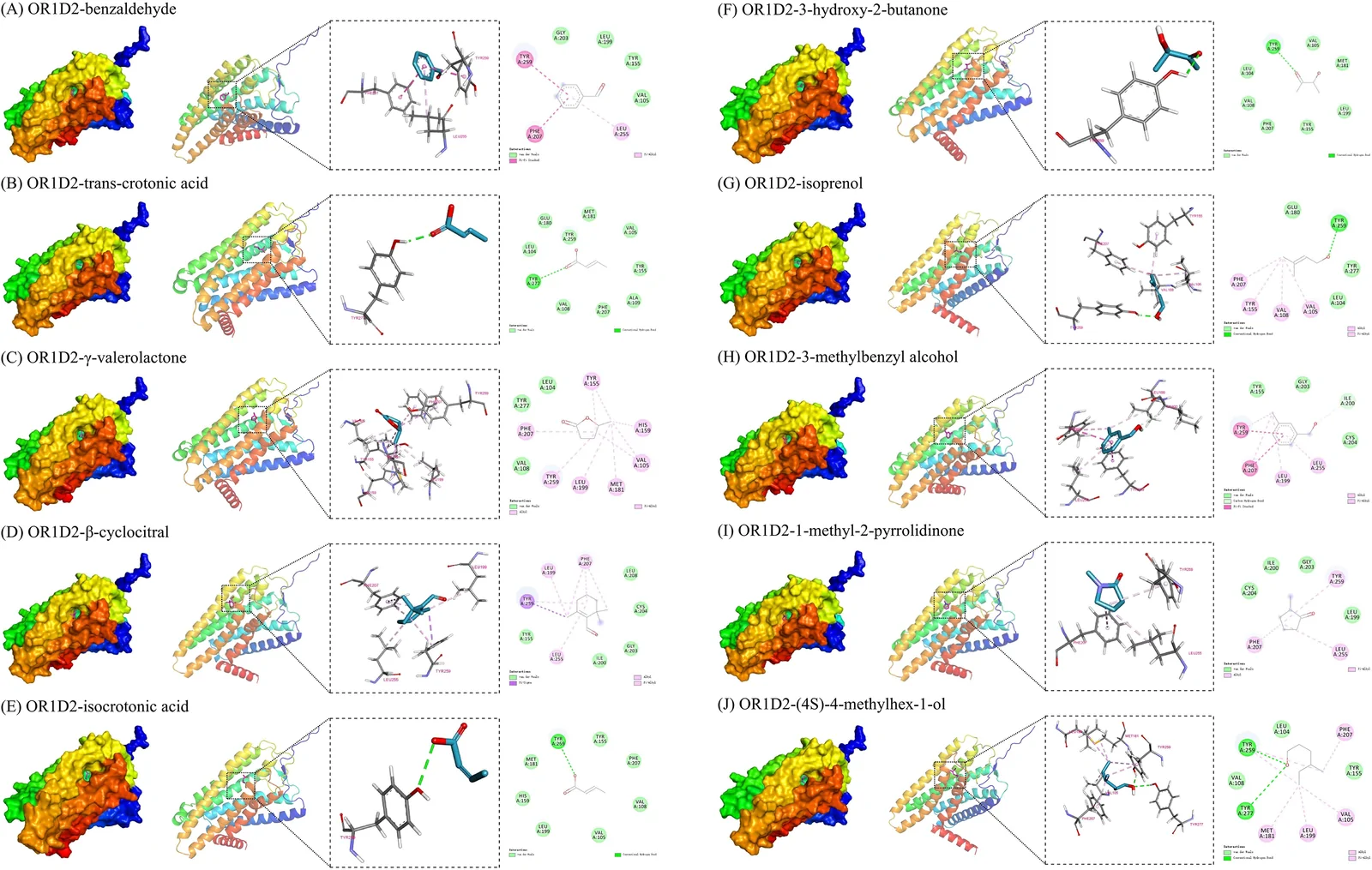
Visualization of molecular docking results between OR1D2 protein and 10 small-molecule ligands.

#### 3.3.2 Molecular dynamics simulation of β-cyclocitral and receptor OR1D2

Given the highest binding affinity of β-cyclocitral among the screened metabolites, we performed a 100-ns MD simulation exclusively on the β-cyclocitral–OR1D2 complex to elucidate the dynamic stability and detailed interaction mechanisms at an atomic level.

##### 3.3.2.1 Trajectory snapshots

After energy minimization and system equilibration, the constructed membrane–protein–small molecule system exhibits the conformation shown in **Figures 8**, **9**. Snapshots from 0 to 100 ns show that the system as a whole maintains a layered/membrane-like aggregated morphology, with no obvious phase separation or structural collapse observed in the purple segments. Throughout the simulation, the green component remains embedded in and partially penetrates the purple matrix, while surrounding segments undergo local rearrangements, indicating stable interactions between the green component and the matrix. The weakened morphological changes from 50 to 100 ns suggest that the membrane–protein–small molecule system remains stable during the simulation.

**Figure 8:**
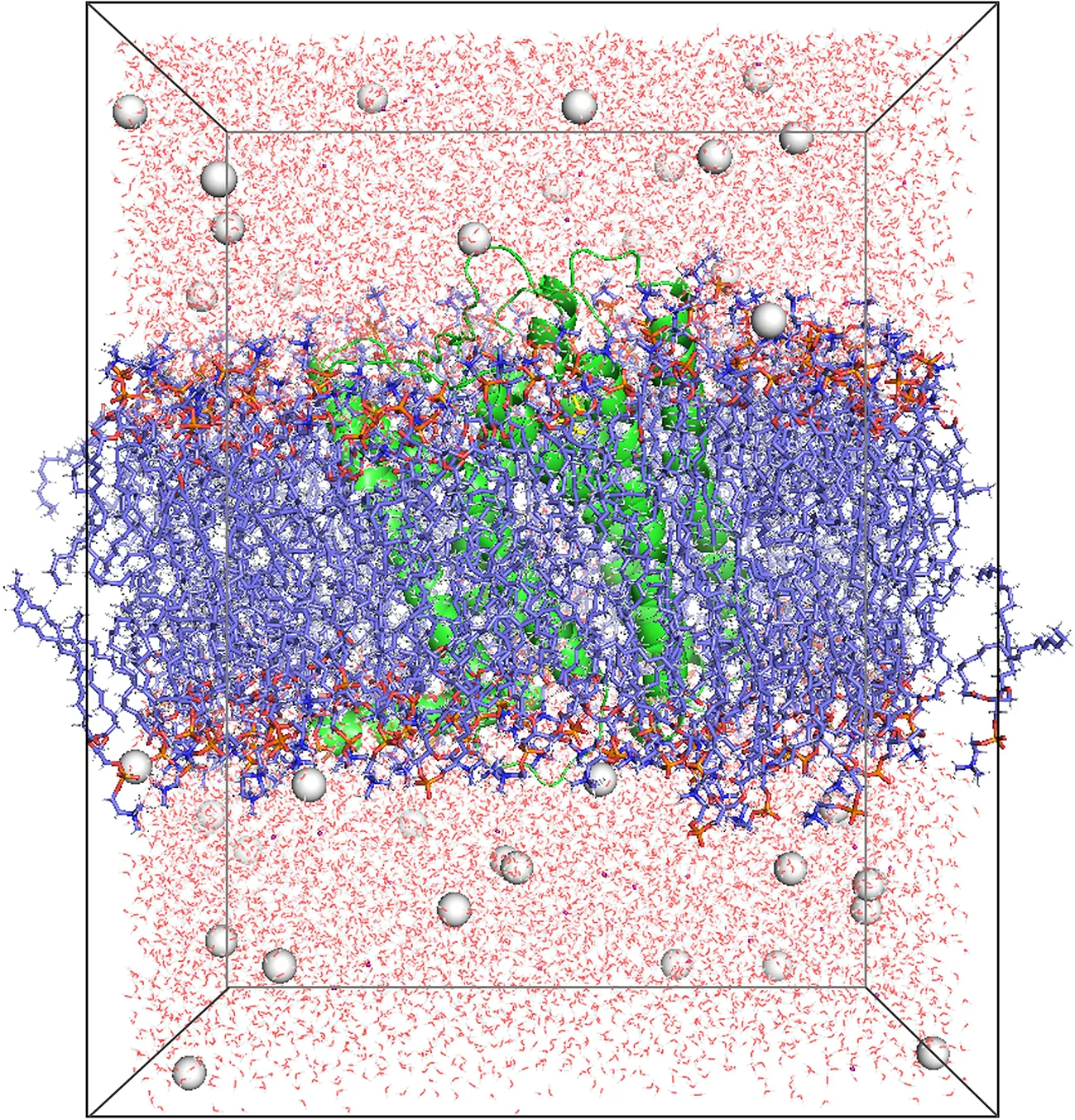
Complex system at equilibrium.

**Figure 9:**
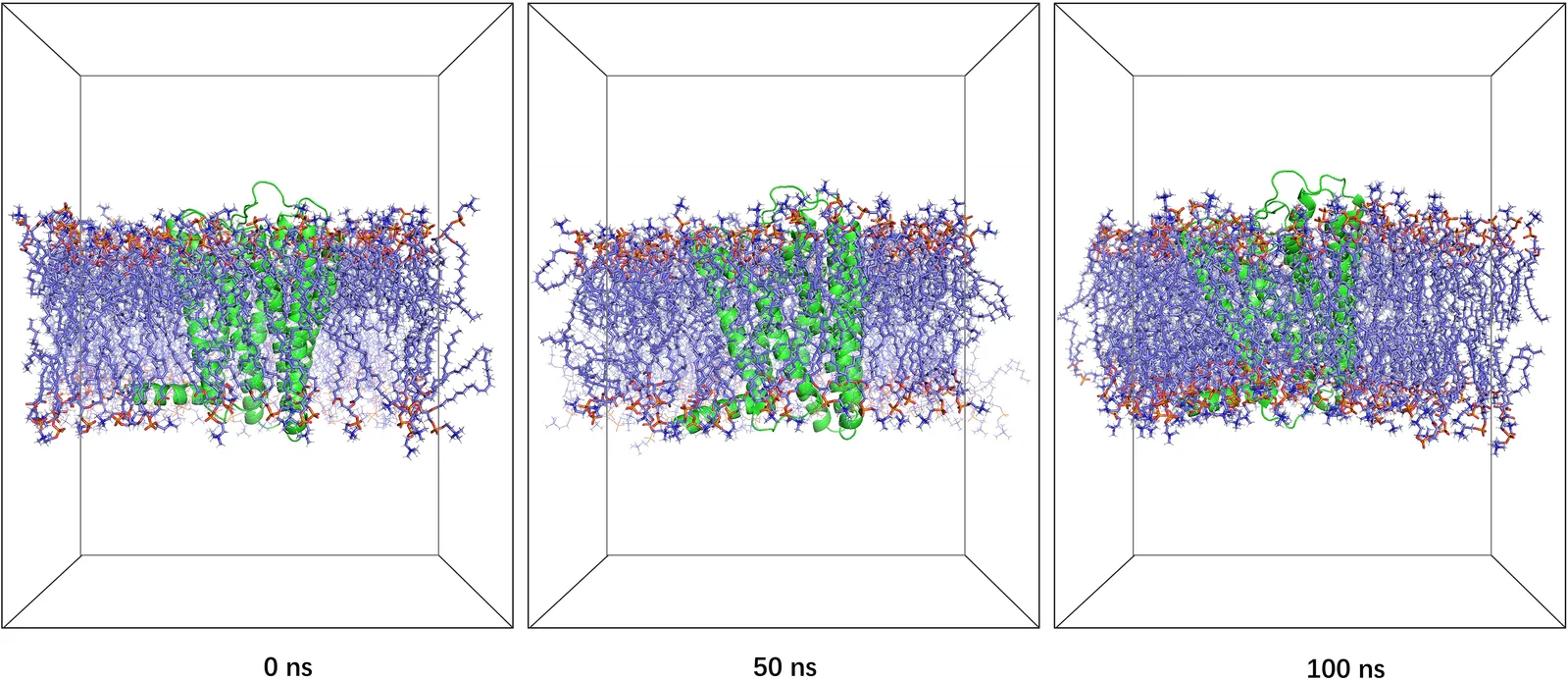
Trajectory snapshot during the simulation.

##### 3.3.2.2 Stability analysis

Surface electrostatic potential analysis (**Figure 10A**) indicates that the small molecule binds within a hydrophobic cavity inside the protein. The overall binding interface is electrically neutral, resulting in only limited hydrogen bonding and electrostatic interactions between the small molecule and the protein. Free energy landscape (FEL) (**Figure 10B**) reveals the existence of two low-energy states in the system, which are primarily attributed to subtle adjustments in local protein conformation and do not significantly affect the overall structural stability of the complex. Processing of the simulation trajectory and conformational superposition (**Figure 10C**) demonstrate a high degree of superposition for the small molecule, confirming that it remains stably positioned within the binding pocket throughout the simulation. Principal Component Analysis (PCA) (**Figure 10E**) shows that the small molecule conformations are concentrated in the phase space, exhibiting a single high-frequency dominant cluster. The hotspot region representing the highest binding probability corresponds to the most stable conformation of the system. Furthermore, the B-Factor map constructed based on RMSF (**Figure 10F**) indicates low flexibility for the amino acids surrounding the binding site, further verifying the binding stability of the complex. In contrast, the relatively high flexibility exhibited by amino acids in the distal regions of the protein suggests that these areas undergo dynamic conformational fluctuations during the simulation.

**Figure 10:**
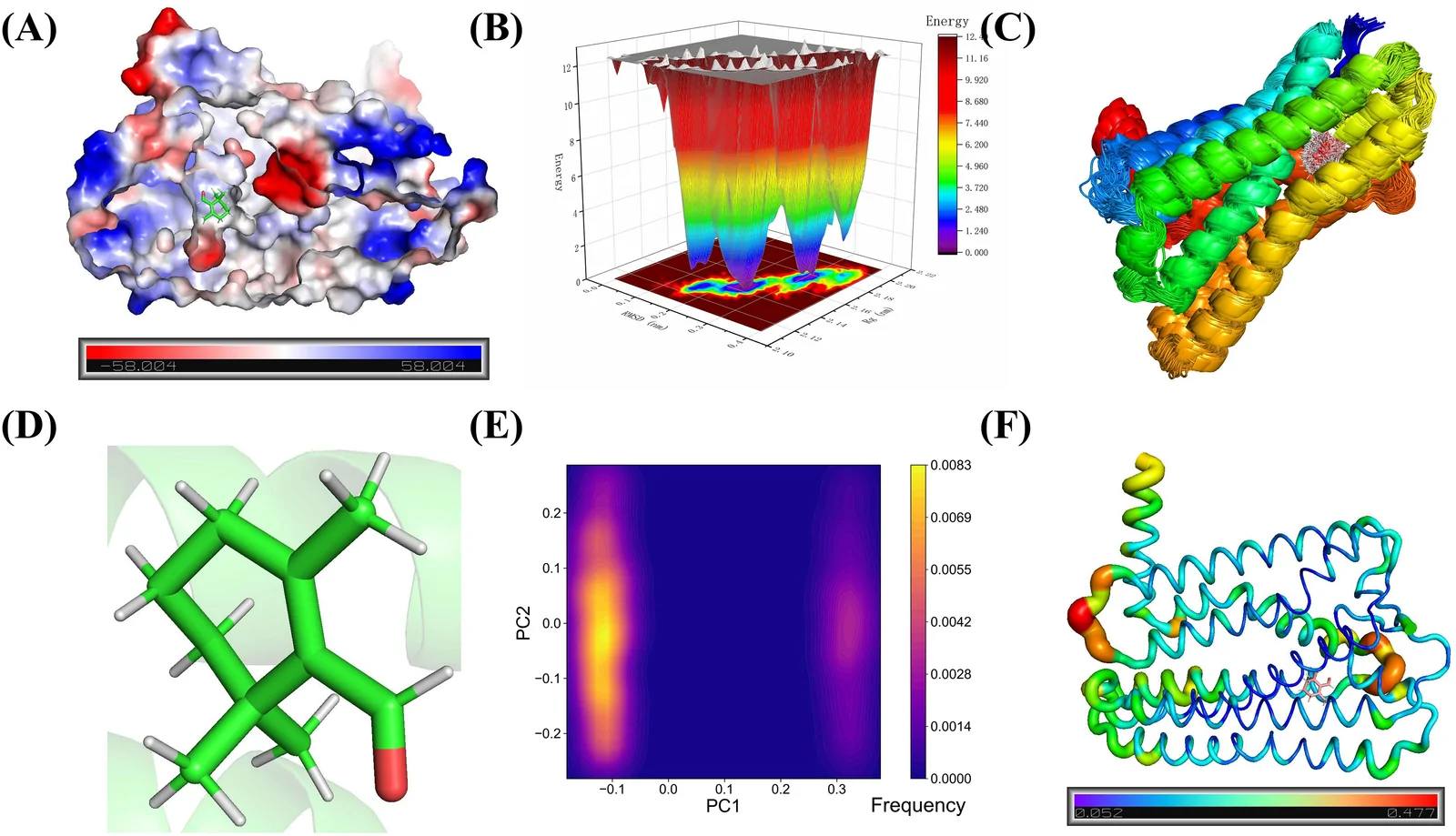
Molecular docking and structural energetic analysis of OR1D2 ligand interactions. **(A)** Electrostatic surface potential; **(B)** free-energy landscape (FEL); **(C)** superposition of simulation conformations; **(D)** ligand binding mode in the binding pocket; **(E)** principal component analysis (PCA); **(F)** B-factor.

Hydrogen bond analysis (**Figure 11A**) shows that only 0–2 hydrogen bonds are formed between the small molecule and the protein. The binding interface is located inside the protein with weak electrostatic interactions, suggesting that the binding is primarily driven by hydrophobic effects. The radius of gyration (Rg) (**Figure 11B**) indicates that the complex structure tends to stabilize after 50 ns without any collapse. The root mean square fluctuation (RMSF) curve (**Figure 11C**) shows overall fluctuations ranging from 0.08 to 0.20 nm; however, significantly high fluctuations are observed for residues 50, 265–275, and the terminal regions, indicating high local flexibility. Root mean square deviation (RMSD) analysis (**Figure 11D**) confirms that the ligand binding pose remains stable within 100 ns, and although the protein undergoes minor adjustments, no dissociation occurs. Both the center-of-mass distance (**Figure 11E**) and the buried solvent accessible surface area (SASA) (**Figure 11F**) converge over time, demonstrating stable binding of the small molecule and a fixed contact area. Binding energy analysis (**Figure 11G**) shows that the sum of van der Waals and electrostatic interactions (Binding) gradually stabilizes, further verifying the binding stability. Energy decomposition (**Figure 11H**) identifies key amino acids such as *TYR-259* and *VAL-105*, which provide the major energy contributions to the binding.

**Figure 11:**
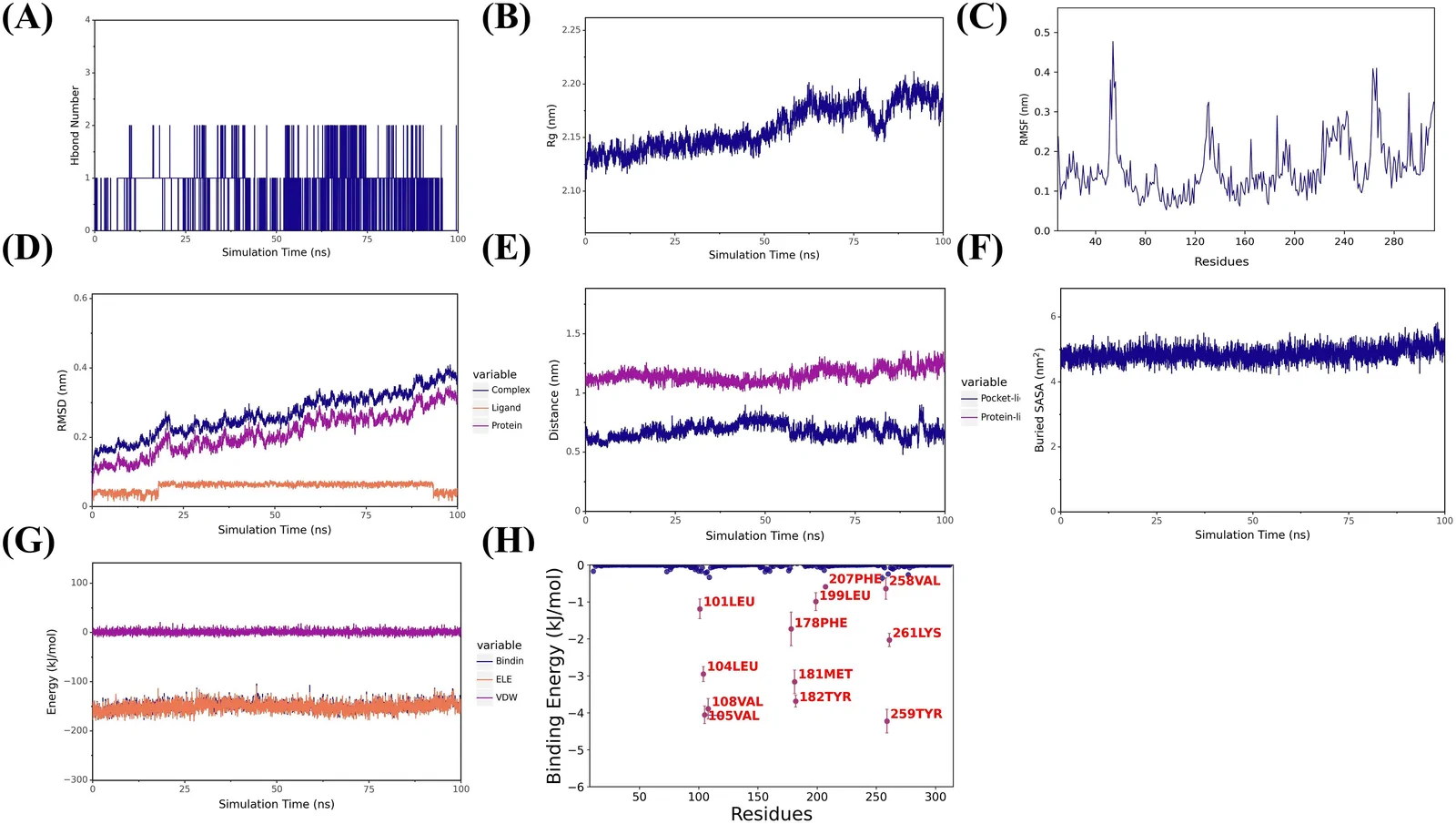
Multidimensional analysis of molecular dynamics simulations. **(A)** Number of hydrogen bonds; **(B)** radius of gyration (Rg); **(C)** root mean square fluctuation (RMSF); **(D)** root mean square deviation (RMSD); **(E)** distance between the protein and ligand binding sites; **(F)** buried solvent accessible surface area (SASA); **(G)** binding free energy components (VDW and ELE) between the protein and ligand; **(H)** per-residue energy contribution.

##### 3.3.2.3 Binding energy analysis

Based on a comprehensive evaluation of RMSD, Rg, Distance, Buried SASA, and interaction energies, the complex trajectory in a stable state was selected. Binding energy-related energy terms were calculated using the molecular mechanics-poisson boltzmann surface area (MM-PBSA) method, as shown in **Table 1**. ΔE_ele_ represents the electrostatic interaction between the small molecule and the protein, ΔE_vdw_ denotes the van der Waals interaction, ΔE_pol_ is the polar solvation energy, ΔE_nonpol_ stands for the non-polar solvation energy, and ΔE_MMPBSA_ is the sum of ΔE_ele_, ΔE_vdw_, ΔE_pol_, and ΔE_nonpol_. The Gibbs binding energy (ΔG_bind_) is the sum of ΔE_MMPBSA_ and -TΔS. Analysis of the table reveals that the van der Waals interaction energy (ΔE_vdw_) is higher than the electrostatic interaction (ΔE_ele_), while the electrostatic interaction is comparable to the hydrophobic interaction (ΔE_nonpol_). Therefore, among the components of the binding energy, van der Waals interactions play a dominant role, whereas electrostatic and hydrophobic interactions play secondary roles. The small molecule exhibits high binding energy and affinity with the protein, with a ΔE_MMPBSA_ of -69.143 ± 0.233 kJ/mol.

**Table 1:** Binding energies and their components at equilibrium state (kJ/mol).

Complex	ΔE_vdw_	ΔE_ele_	ΔE_pol_	ΔE_nonpol_	ΔE_MMPBSA_	–TΔS	ΔG_bond_*
Protein-ligand	-97.986 ± 0.414	-5.088 ± 1.466	47.541 ± 1.259	-13.61 ± 0.069	-69.143 ± 0.233	9.077 ± 0.515	-60.066 ± 0.411

*ΔG_bond_ = ΔE_vdw_ + ΔE_ele_ + ΔE_pol_ + ΔE_nonpol_ – TΔS.

## 4 Conclusions and discussion

Maturity critically determines tobacco leaf quality by shaping microstructure and metabolism. Using SEM and untargeted metabolomics, middle leaves of Yunyan 87 were analyzed across underripe (QS), mature (GS), and overripe (CS) stages. Mature and overripe leaves showed more stomata, elliptical (vs. broadly elliptical) guard cells, larger intercellular spaces, and striated/ridged surfaces. Metabolite counts were 226 (QS/GS) and 233 (CS), with 10 differential metabolites identified. Mature leaves had higher aroma compounds (γ-valerolactone, benzaldehyde, β-cyclocitral, 3-hydroxy-2-butanone, isoprenol) and lower off-flavor compounds (isocrotonic acid, 1-methyl-2-pyrrolidinone) than the other stages. Harvesting at the mature stage thus optimizes leaf quality.

The aroma quality of tobacco leaves is one of the key indicators for evaluating their overall quality. β-Cyclocitral exists in a free state in tobacco leaves and serves as a mature aroma component. Under processing conditions, it can be further oxidized into derivatives such as β-cyclocitric acid (Ramel et al., 2012). 3-Hydroxy-2-butanone exhibits intense creamy and fatty aromas (Han et al., 2024). γ-Valerolactone exhibits vanillin and coconut fragrances with warm, sweet herbal characteristics (Wang et al., 2024; Song et al., 2025). Benzaldehyde features a distinctive almond aroma, complemented by fruity notes particularly cherry which contribute to its widespread application in the food and flavor industries (Hong et al., 2023; Li et al., 2025). PubChem describes 1-methyl-2-pyrrolidinone (NMP) as a colourless liquid with a faint amine-like or mild fishy odor. In contrast, the odor characteristics of isocrotonic acid were not experimentally validated in this study; instead, they were qualitatively inferred based on its Globally Harmonized System of Classification and Labelling of Chemicals (GHS) hazard statements descriptions of sour, pungent, and irritating notes.

These findings reveal significant differences in the aroma quality of tobacco leaves across varying maturity stages. Studies on K326 middle leaves have demonstrated that harvesting at the optimal maturity stage yields the best outcomes in terms of fresh leaf maturity traits, cured leaf quality, and sensory evaluation (Dai et al., 2024). Underripe leaves exhibit pronounced green and astringent flavors with relatively weak aroma. As maturity progresses, the accumulation of aromatic compounds increases substantially, resulting in a richer, more full-bodied tobacco aroma and a smoother, sweeter taste with diminished irritation. Mature leaves exhibit unique aromatic profiles defined by balanced richness and harmony, often characterized by multiple fragrance components, including floral, fruity, and nutty notes (Shi et al., 2024). In contrast, overripe and fully ripe leaves may exhibit aroma deterioration or off-odors, leading to quality decline, likely caused by the decomposition of certain aromatic compounds and the accumulation of undesirable odorants. These findings suggest that selecting optimal maturity for tobacco harvesting and drying is essential for improving leaf quality. The molecular docking analysis revealed significant variations in the binding affinities of the ten differential metabolites to OR1D2, with β-cyclocitral exhibiting the highest binding capacity. Given this superior affinity, we performed MD simulations and MM-PBSA binding energy decomposition exclusively on the β-cyclocitral-OR1D2 complex to elucidate the dynamic interaction mechanism at an atomic level. The results demonstrated that β-cyclocitral forms a stable complex with the receptor, where​ van der Waals interactions serve as the primary driving force, supplemented by electrostatic and hydrophobic interactions. It is important to note that these energetic details are specific to β-cyclocitral; caution should be exercised when extrapolating these dynamic interaction characteristics to the other nine ligands. Future studies extending MD simulations to additional high-affinity ligands are warranted to comprehensively characterize the dynamic binding mechanisms of tobacco flavor compounds with OR1D2.

In the future, appropriate regulatory measures including cultivation management, harvest timing and methods, and drying techniques are essential for ensuring optimal leaf maturity and quality. Tobacco growers and producers should implement scientifically validated strategies tailored to field conditions to regulate leaf maturity and improve product quality. Furthermore, in-depth research is required to clarify the relationship between leaf maturity and quality, and to develop more precise and efficient regulation technologies that can provide technical support for advancing the tobacco industry.

This study employed molecular docking and 100 ns molecular dynamics simulations to predict the binding modes between key differential flavor compounds and OR1D2. However, these in silico findings remain to be experimentally validated through affinity assays such as surface plasmon resonance (SPR) or isothermal titration calorimetry (ITC); future work will therefore encompass *in vitro* receptor binding assays and sensory evaluations to empirically substantiate the olfactory contributions of these compounds. Furthermore, although HS-SPME-GC-MS provides high sensitivity for volatile profiling, it is liable to underestimate or overlook non-volatile flavor precursors that critically influence the final aroma during leaf curing and aging. Subsequent investigations will integrate complementary platforms such as liquid chromatography–mass spectrometry (LC-MS) to achieve more comprehensive metabolomic coverage. Lastly, while this work identified mature-stage differential metabolites and predicted their OR1D2 binding affinities, the absence of actual sensory quality assessments, electronic nose analyses, or human panel validation implies that observed correlations between metabolite abundance, receptor binding energy, and the ultimate sensory experience remain correlative rather than causative, necessitating further empirical corroboration.

## Data Availability

The data generated in this study are available in the figshare repository: https://doi.org/10.6084/m9.figshare.34296555.
